# Modulation of microtubule assembly by the HIV-1 Tat protein is strongly dependent on zinc binding to Tat

**DOI:** 10.1186/1742-4690-5-62

**Published:** 2008-07-09

**Authors:** Caroline Egelé, Pascale Barbier, Pascal Didier, Etienne Piémont, Diane Allegro, Olivier Chaloin, Sylviane Muller, Vincent Peyrot, Yves Mély

**Affiliations:** 1 Université Louis Pasteur, Strasbourg 1, Institut Gilbert Laustriat, CNRS, UMR 7175, Département Photophysique des Interactions Biomoléculaires, Faculté de Pharmacie, 74, Route du Rhin, 67401, Illkirch, Cedex, France; 2 Aix-Marseille Université, INSERM UMR 911, Centre de Recherche en Oncologie biologique et en Oncopharmacologie, Faculté de Pharmacie, 27, Boulevard Jean Moulin, 13385, Marseille, Cedex 5, France; 3 CNRS UPR 9021, Institut de Biologie Moléculaire et Cellulaire, 15 rue René Descartes, Strasbourg, France

## Abstract

**Background:**

During HIV-1 infection, the Tat protein plays a key role by transactivating the transcription of the HIV-1 proviral DNA. In addition, Tat induces apoptosis of non-infected T lymphocytes, leading to a massive loss of immune competence. This apoptosis is notably mediated by the interaction of Tat with microtubules, which are dynamic components essential for cell structure and division. Tat binds two Zn^2+ ^ions through its conserved cysteine-rich region *in vitro*, but the role of zinc in the structure and properties of Tat is still controversial.

**Results:**

To investigate the role of zinc, we first characterized Tat apo- and holo-forms by fluorescence correlation spectroscopy and time-resolved fluorescence spectroscopy. Both of the Tat forms are monomeric and poorly folded but differ by local conformational changes in the vicinity of the cysteine-rich region. The interaction of the two Tat forms with tubulin dimers and microtubules was monitored by analytical ultracentrifugation, turbidity measurements and electron microscopy. At 20°C, both of the Tat forms bind tubulin dimers, but only the holo-Tat was found to form discrete complexes. At 37°C, both forms promoted the nucleation and increased the elongation rates of tubulin assembly. However, only the holo-Tat increased the amount of microtubules, decreased the tubulin critical concentration, and stabilized the microtubules. In contrast, apo-Tat induced a large amount of tubulin aggregates.

**Conclusion:**

Our data suggest that holo-Tat corresponds to the active form, responsible for the Tat-mediated apoptosis.

## Background

Human Immunodeficiency Virus type 1 (HIV-1) infection is characterized by a massive depletion of CD4+ T cells that leads to the loss of immune competence [1,2]. This is in part mediated by the HIV-1 Tat protein, which is produced by HIV-infected cells and is efficiently taken up by the neighboring cells [3-5]. Tat is an 86 to 106-amino acid-long protein whose primary role is to transactivate the transcription of the HIV-1 proviral DNA from the long terminal repeat (LTR) by binding to the nascent TAR (Trans-Acting Responsive element) RNA sequence [6-8].

In addition, extracellular Tat shows many additional functions, which contribute to the AIDS syndrome. In particular, Tat induces the apoptosis of macrophages and cytotoxic T-lymphocytes by several mechanisms [9]. These different pathways include the up-regulation of Fas ligand [10], the down-regulation of cellular genes encoding for superoxide-dismutase [11] and manganese-dependent superoxide dismutase [12], and the activation of cyclin dependent kinases [13]. Another mechanism of Tat-mediated apoptosis involves microtubules [14-16], which are polymers of α- and β-tubulin dimers involved in numerous cellular functions such as mitosis, cell motility, or intracellular traffic. Tat is thought to interact in the cytoplasm with tubulin dimers and microtubules through a four-amino acid subdomain (amino acids 36 to 39) within its highly conserved 13-amino acid core region (amino acids 36 to 48) [15]. These interactions alter the microtubule dynamics [14-17], inducing the mitochondrial pathway of cellular apoptosis [15,18] as well as neuronal cytoskeletal changes leading to the neurodegenerative diseases associated with AIDS [17].

Tat has been shown to bind two Zn^2+ ^ions *in vitro *[19-21] through its conserved cysteine-rich domain (residues 22–37), which is well exposed to solvent [22,23]. However, the role of zinc in the structure and functions of Tat is still debated. Indeed, while Tat has been proposed to form a metal-linked dimer with zinc ions bridging the cysteine-rich regions from each monomer [19], Tat was described by others to remain monomeric in the presence of zinc [6,21,24]. Moreover, while the binding of zinc was reported to be dispensable for the binding of Tat to the TAR sequence [19] and for the role of Tat in the transactivation step [24], it was shown to be required for the interaction with T1 cyclin, essential for the transactivation of proviral DNA transcription [25]. Interestingly, zinc binding has also been shown to be critical for Tat-induced apoptosis [26]. Since apoptosis mediated by Tat partly relies on the interaction of Tat with tubulin [14-17], we hypothesized that zinc binding might play a role in the modulation by Tat of the microtubule dynamics.

Thus, in order to get insight in the role of zinc in the molecular mechanism of Tat-induced apoptosis, we analyzed the conformations of the apo-form and zinc-bound form of Tat, and studied the interaction of the two forms of Tat with tubulin. The 86-aa-long Tat protein was synthesized by solid-phase chemistry and was shown to be highly pure and biologically active [27]. Using fluorescence correlation spectroscopy (FCS) and time-resolved fluorescence spectroscopy, the two forms were found to be monomeric and poorly folded, and to differ by local conformational changes in the vicinity of the cysteine-rich region. Moreover, using turbidity measurements and electron microscopy, both forms were found to promote tubulin assembly, but only the holo-Tat decreased the tubulin critical concentration and promoted cold stable microtubules. These observations were correlated with the different binding modes of the two Tat forms on tubulin dimers.

## Methods

### Chemical synthesis of Tat protein from HIV-1 Lai

The full-length Tat protein from HIV-1 Lai strain (^1^MEPVDPRLEPWKHPGSQPKTACTTCYCKKCCFHCQVCFTTKAL

GISYGRKKRRQRRRPPQGSQTHQVSLSKQPTSQPRGDPTGPKE^86^) was chemically synthesized and purified as described previously [27]. Tat-RhB was synthesized using the same strategy. Tat samples were stored lyophilized at -20°C to prevent oxidation. The thirteen aa-long Tat(36–48) peptide was synthesized by NeoMPS (France).

### Treatments of Tat proteins

Apo-Tat was used four hours after dissolution in the appropriate buffer. In these conditions, apo-Tat was spontaneously oxidized with the formation of essentially intramolecular disulfide bridges [24]. Reduced apo-Tat was obtained by adding 1 mM TCEP (Tris (2-carboxyethyl) phosphine hydrochloride), which keeps the -SH groups in a reduced form, to the buffer. Holo-Tat was prepared by addition of two molar equivalents of zinc (ZnSO_4_). For fluorescence measurements, Tat proteins were dissolved in 50 mM Hepes buffer, pH7.5. For FCS measurements, the 50 mM Hepes buffer pH7.5 contained also 0.05% (v/v) of IGEPAL CA-630 to limit Tat adsorption to the walls of the Lab-Tek wells. For the other techniques, Tat proteins were dissolved in 20 mM sodium phosphate (NaPi) buffer, pH6.5 to monitor Tat-tubulin interactions. Tat concentration was determined on a Cary 400 spectrophotometer (Varian, Australia) by using an extinction coefficient of 8,300 M^-1^cm^-1 ^at 280 nm. For Tat-RhB, we used an extinction coefficient of 65,950 M^-1^cm^-1 ^at 555 nm.

### Determination of Tat sulfhydryl concentration

The oxidation of Tat was monitored by Ellman's method [28]. The titration of the sulfhydryl groups was performed with DTNB (5,5'-dithiobis(2-nitrobenzoic acid), in the presence of EDTA. The concentration of the free -SH groups of Tat was monitored by measuring the absorbance at 412 nm with a Cary 4000 spectrophotometer, using ε_412 nm _= 13,600 M^-1 ^cm^-1 ^[29].

### FCS setup and data analysis

FCS measurements were performed on a two-photon platform including an Olympus IX70 inverted microscope, as described previously [30,31]. Two-photon excitation at 850 nm is provided by a mode-locked Tsunami Ti:sapphire laser pumped by a Millenia V solid state laser (Spectra Physics, U.S.A.). The measurements were carried out in an eight-well Lab-Tek II coverglass system, using a 400-μL volume per well. The focal spot was set about 20 μm above the coverslip. The normalized autocorrelation function, *G(τ) *was calculated online by an ALV-5000E correlator (ALV, Germany) from the fluorescence fluctuations, *δF(t)*, by G(τ) = <δF(t)δF(t+τ)>/<F(t)>^2 ^where <*F(t)*> is the mean fluorescence signal, and *τ *is the lag time. Assuming that Tat-Rhodamine B (Tat-RhB) undergoes triplet blinking and diffuses freely in a Gaussian excitation volume, the correlation function, *G(τ)*, calculated from the fluorescence fluctuations was fitted according to [32]:

(1) G(τ)=1N(1+ττd)−1(1+1s2ττd)−12(1+(ft1−ft)exp⁡(−τ/τt))

where *τ*_*d *_is the diffusion time, *N *is the mean number of molecules within the sample volume, *S *is the ratio between the axial and lateral radii of the sample volume, *f*_*t *_is the mean fraction of fluorophores in their triplet state and *τ*_*t *_is the triplet state lifetime. The excitation volume is about 0.3 μm^3 ^and *S *is about 3 to 4. Using carboxytetramethylrhodamine (TMR) in water as a reference (D_TMR _= 2.8× 10^-6 ^cm^2^·s^-1^) [33], the diffusion coefficient, *D*_*exp*_, of the labeled peptide was calculated by: D_exp_=D_TMR _× τ_d(TMR)_/τ_d(Tat) _where *τ*_*d*(*TMR*) _and *τ*_*d*(*Tat*) _are the measured correlation times for TMR and Tat-RhB, respectively. Typical data recording times were 10 min.

### Time-resolved fluorescence measurements

Time-resolved fluorescence measurements were performed with the time-correlated, single-photon counting technique, as previously described [34,35]. The excitation and emission wavelengths for Trp residues were set at 295 nm and 350 nm, respectively. For lifetime measurements, the polarizer in the emission path was set at the magic angle (54.7°). For time-resolved anisotropy measurements, this polarizer was set at the vertical position. *I*_⊥ _*(t) *and *I*_//_*(t) *were recorded alternatively every 5 s, by using the vertical polarization of the excitation beam with and without the interposition of a quartz crystal that rotates the beam polarization by 90°. Time-resolved data analysis was performed by the maximum entropy method using the Pulse5 software [36]. For the analysis of the fluorescence decay, a distribution of 200 equally spaced lifetime values on a logarithmic scale between 0.01 and 10 ns was used. The anisotropy decay parameters were extracted from both *I*_⊥ _(*t*) and *I*_//_(*t*). The anisotropy at any time *t *is given by:

(2) $$r\left(t\right)=r_{0}\sum_{i}\beta_{i}e^{-t/\theta_{i}}$$

where *r*_0 _is the fundamental anisotropy, and *β*_*i *_corresponds to the fractional amplitude, which decays with the correlation time *θ*_*i*_.

### Tubulin purification

Tubulin was purified from lamb brains by ammonium sulfate fractionation and ion exchange chromatography. The protein was stored in liquid nitrogen and prepared as previously described [37-39]. Protein concentrations were determined spectrophotometrically with an extinction coefficient of ε_275nm _= 1.07 L.g^-1^·cm^-1 ^in 0.5% SDS in neutral aqueous buffer, or with ε_275 nm _= 1.09 L.g^-1^·cm^-1 ^in 6 M guanidine hydrochloride.

### Sedimentation velocity

Experiments were performed in PG buffer (20 mM NaPi, 10 μM GTP, pH6.5), at 20°C (non-assembly conditions). Experiments were carried out at 40,000 rpm in a Beckman Optima XL-A analytical ultracentrifuge equipped with absorbance optics, using an An55Ti rotor and 12 mm aluminum double-sector centerpieces. Tubulin solutions (5 μM), in the absence or in the presence of Tat were centrifuged and the absorbance was recorded in the continuous mode at 290 nm to minimize the contribution of Tat absorption. The apparent sedimentation coefficients were determined using the SEDFIT program [40] and corrected to the standard conditions by the SEDNTERP program (retrieved from the RASMB server).

### Microtubule formation

The classical buffer used to measure microtubule assembly is the PEMG buffer: 20 mM NaPi, 1 mM EGTA (ethylene glycol tetraacetic acid), 10 mM MgCl_2_, 0.1 mM GTP, and 3.4 M glycerol, pH 6.5 [41]. We performed our experiments in PMG buffer without EGTA, to avoid chelating zinc from Tat. Various concentrations of Tat were mixed with 15 μM tubulin (assembly conditions above the critical concentration Cr to obtain tubulin polymerization) or 6 μM tubulin (assembly conditions under the Cr) at 4°C on ice. The assembly reactions were started by warming the samples to 37°C in a 0.2 × 1 cm cell, and the polymer formation was monitored by turbidimetry at 350 nm using a thermostated Beckman DU7400 spectrophotometer.

### Critical concentration determination

Holo-Tat (8 μM) was added to tubulin samples (concentrations ranging from 0.3 to 25 μM tubulin) in PMG buffer. The samples were incubated for 40 min at 37°C and centrifuged for 30 min at 50,000 rpm with a TL100 Beckman ultracentrifuge in a prewarmed TLA 100.2 rotor. Supernatants were carefully removed by aspiration. The tubulin concentration in the supernatant, which corresponds to Cr, was measured spectrofluorometrically, by comparison with a calibration curve of the fluorescence emission as a function of known tubulin concentrations. Fluorescence emission spectra were recorded on a FluoroMax spectrofluorometer (Jobin Yvon) with an excitation wavelength of 295 nm. A control with holo-Tat alone (8 μM) was done in parallel following the same procedure in order to subtract holo-Tat fluorescence from the samples.

### Electron Microscopy

Samples were adsorbed onto 200 meshes, Formvar carbon-coated copper grids, stained with 2% (w/v) uranyl acetate, and blotted to dryness. Grids were observed using a JEOL JEM-1220 electron microscope operated at 80 kV. For assembly assays at 37°C, to ensure that the polymers do not disassemble, grids were prepared in a thermostated room at 37°C.

## Results

### Zinc binding prevents Tat oxidation

**Figure 1:**
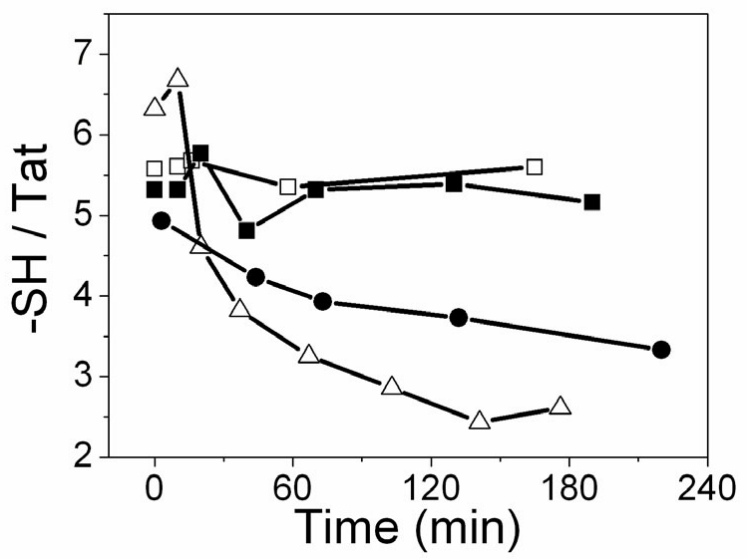
**Effect of zinc binding on Tat oxidation**. The number or free -SH groups per Tat molecule was measured according to the Ellman reaction. Tat in NaPi 20 mM buffer, pH6.5 (●), or in Hepes buffer 50 mM, pH7.5, in the absence (△), or in the presence of 2 (■) or 5 (□) zinc equivalents.

As a first step, we measured the effect of zinc binding on Tat oxidation. To this end, we monitored with time the number of free -SH groups per molecule of Tat. At pH7.5 in the absence of zinc, oxidation occurs rapidly, as well documented [21]. Five out of the seven -SH groups were oxidized within three hours (Fig. 1). Since Tat-tubulin interaction was investigated at pH6.5, we also measured the oxidation of Tat at this pH. Oxidation was slower than that at pH7.5, but nevertheless three out of the seven -SH groups were oxidized after four hours. In contrast, two equivalents of zinc preserved Tat from oxidation since five out of seven -SH groups remained in their reduced form, even after more than 24 hours (data not shown). There was no difference with five equivalents of zinc, suggesting that Tat is saturated with two equivalents of zinc. This is in agreement with mass spectrometry data, which showed the disappearance of apo-Tat when two zinc equivalents were added (data not shown).

### Zinc binding induces a local folding of Tat

**Figure 2:**
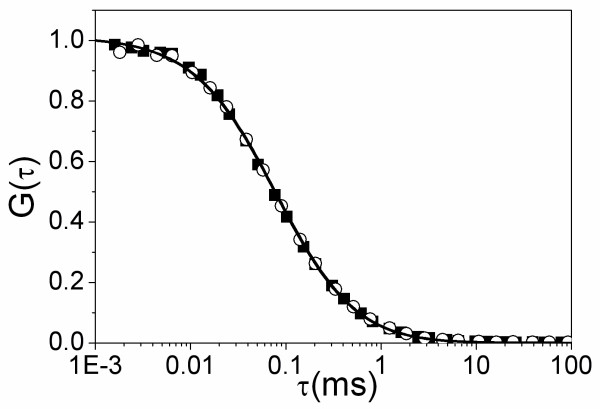
**Effect of zinc on Tat-RhB diffusion, as monitored by FCS**. The normalized autocorrelation curves were recorded with 1 μM apo-Tat-RhB (○) or holo-Tat-RhB (■) in Hepes buffer 50 mM, 0.05% IGEPAL CA-230, pH7.5, at 20°C. The continuous lines are fits to the experimental points with Equation 1.

In a next step, we characterized the effect of zinc on the structure of Tat. To this end, we first performed fluorescence correlation spectroscopy (FCS) using Tat labeled at its N-terminus by rhodamine B (Tat-RhB). The autocorrelation curves of apo-Tat-RhB and holo-Tat-RhB were indistinguishable (Fig. 2). Their diffusion constants were 1.46(± 0.05) × 10^-6 ^cm^2^s^-1 ^and 1.38(± 0.08) × 10^-6 ^cm^2^s^-1^, respectively, in excellent agreement with the theoretical diffusion constant (*D*_*th *_= 1.44 × 10^-6 ^cm^2^s^-1^) calculated from the Stokes-Einstein equation for the diffusion of a sphere with the molecular mass of the Tat protein and 30% hydration. This suggests that both protein forms are monomeric with a nearly spherical shape. Moreover, the identical brightness (5.1 ± 0.1 kHz/molecule) of the two Tat forms confirmed that they exhibit the same oligomeric state. Interestingly, the monomeric state of both Tat forms was further substantiated by mass spectrometry (data not shown).

Then, we performed steady-state and time-resolved fluorescence measurements, by monitoring the signal of Trp^11^, which is a strictly conserved residue among Tat variants [22,23]. Steady-state fluorescence results (data not shown) showed that apo-Tat and holo-Tat displayed their maximum emission wavelength at 346 nm, consistent with a well exposed Trp residue [42]. The fluorescence intensity decay of apo-Tat was characterized by four lifetimes ranging from 0.21 ns to 4.5 ns, with comparable populations (Table 1). Addition of two equivalents of zinc resulted in a significant increase of the long-lived lifetime from 4.5 ns to 5.1 ns. In contrast, the other lifetimes as well as the amplitudes associated with the various lifetimes were only marginally affected by the binding of zinc. This suggests that the environment of Trp^11 ^is only moderately modified by the binding of zinc ions.

**Table 1:** Fluorescence intensity decay parameters of apo-Tat and holo-Tat^*a*^

	*τ*_1 _(ns)	*α*_1 _(%)	*τ*_2 _(ns)	*α*_2 _(%)	*τ*_3 _(ns)	*α*_3 _(%)	*τ*_4 _(ns)	*α*_4 _(%)	<τ> (ns)
Apo-Tat	0.21 ± 0.03	25 ± 2	1.35 ± 0.01	35 ± 3	2.60 ± 0.20	19 ± 1	4.5 ± 0.2	21 ± 5	1.96 ± 0.08
Holo-Tat	0.22 ± 0.05	18 ± 4	1.30 ± 0.20	37 ± 3	2.79 ± 0.09	25 ± 3	5.1 ± 0.2	20 ± 3	2.24 ± 0.07

^*a *^Experiments were performed with 1.5 μM Tat proteins in 50 mM Hepes buffer, pH7.5, at 20°C. The lifetimes, τ_*i*_, and relative amplitudes, α_*i*_, are expressed as means for at least three independent experiments. The mean lifetimes were calculated with: ⟨τ⟩ = ∑α_*i*_*τ*_*i*_. The excitation and emission wavelengths for Trp were set at 295 nm and 350 nm, respectively.

Fluorescence anisotropy decays showed that both forms were characterized by two correlation times (Table 2). The short correlation time was about 0.25 ns for both forms and can be assigned to the local motion of the Trp residue [42]. The long correlation time was 2 ns for apo-Tat and was thus markedly lower than the 4.1 ns theoretical value expected for the tumbling motion of a sphere with the molecular mass of Tat and 30% hydration [42]. The long correlation time likely describes the segmental motion of a domain, which includes the Trp residue. A significant increase of this long correlation time (from 2 ns to 2.8 ns) was observed with addition of zinc, indicating a significant slowing down of the motion of the Trp-containing domain. This slowing down is likely related to a zinc-induced folding of the Cys-rich sequence (residues 22–37), which is close to the Trp^11 ^residue.

**Table 2:** Fluorescence anisotropy decay parameters of apo-Tat and holo-Tat^*a*^

	*θ*_1 _(ns)	*β*_1 _(%)	*θ*_2 _(ns)	*β*_2 _(%)
Apo-Tat	0.28 ± 0.03	42 ± 3	2.0 ± 0.2	58 ± 3
Holo-Tat	0.24 ± 0.07	43 ± 6	2.8 ± 0.4	57 ± 6

^*a *^Experimental conditions were as in Table 1. The correlation times, *θ*_*i*_, and relative amplitudes, *β*_*i*_, are expressed as means for at least three experiments.

Noticeably, no significant changes in the steady-state and time-resolved fluorescence parameters of the apo-Tat were observed in the presence of TCEP that keeps the -SH groups in a reduced form. This indicates that the intramolecular disulfide bridges in the oxidized form of apo-Tat do not significantly affect the environment and the local motion of Trp^11 ^as well as the segmental motion of the Trp-containing domain.

### Zinc binding to Tat promotes discrete Tat-tubulin complexes under non-assembly conditions

**Figure 3:**
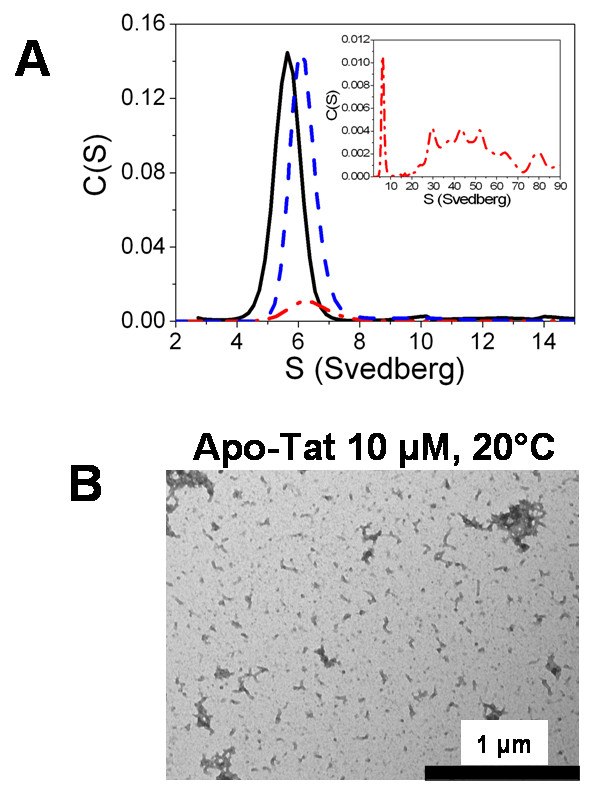
**Zinc binding to Tat promotes discrete Tat-tubulin complexes under non-assembly conditions**. A. Characterization of Tat-tubulin interaction by analytical ultracentrifugation, in PG buffer. Continuous sedimentation coefficient distribution C(S) of tubulin (5 μM) in the absence (black solid line), or in the presence of 10 μM holo-Tat (blue dashed line) or 10 μM apo-Tat (red dotted line). Inset: Full range C(S) of tubulin (5 μM) in the presence of 10 μM apo-Tat (red dotted line). Tat contributed to less than 10% of the signal. B. Electron micrograph of 5 μM tubulin in the presence of 10 μM apo-Tat, in PG buffer.

We first investigated the interaction of Tat with tubulin dimers at 20°C in 20 mM NaPi, 10 μM GTP, pH6.5 (PG buffer). This buffer normally allows neither the association of tubulin nor microtubule assembly at a tubulin concentration ≤ 5 μM [43]. Analytical ultracentrifugation (AUC) was used to characterize the binding of both apo-Tat and holo-Tat to tubulin dimers. Control tubulin (5 μM) was found to sediment as a single species, as indicated by the single Gaussian distribution of the continuous sedimentation coefficient, C(S) (Fig. 3A) centered at 5.64 ± 0.01 S, in line with the standard value $S_{20,W}^{0}$ of 5.8 S [39]. Control experiments with zinc sulfate at concentrations up to 20 μM, corresponding to the total concentration of zinc used in the holo-Tat samples, did not change the apparent sedimentation coefficient (*S*_*apparent*_) of tubulin and its corresponding area (data not shown). In contrast, the *S*_*apparent *_of tubulin in the presence of 10 μM holo-Tat increased to 6.12 ± 0.01 S, suggesting a direct interaction of the holo-Tat with tubulin dimers. In the presence of apo-Tat at the same concentration (10 μM), the *S*_*apparent *_value of tubulin also increased and reached a value of 6.29 ± 0.02 S. However, the area of the corresponding peak drastically decreased in favor of a distribution of *S*_*apparent *_values ranging from 20 to 90 S (Fig. 3A inset), suggesting the formation of tubulin oligomers. Electron microscopy of the tubulin/apo-Tat samples (Fig. 3B) showed the presence of small particles, consistent with the formation of oligomers, which are absent in the control and the tubulin- holo-Tat samples (data not shown).

### Holo-Tat promotes and stabilizes microtubules under assembly-conditions

**Figure 4:**
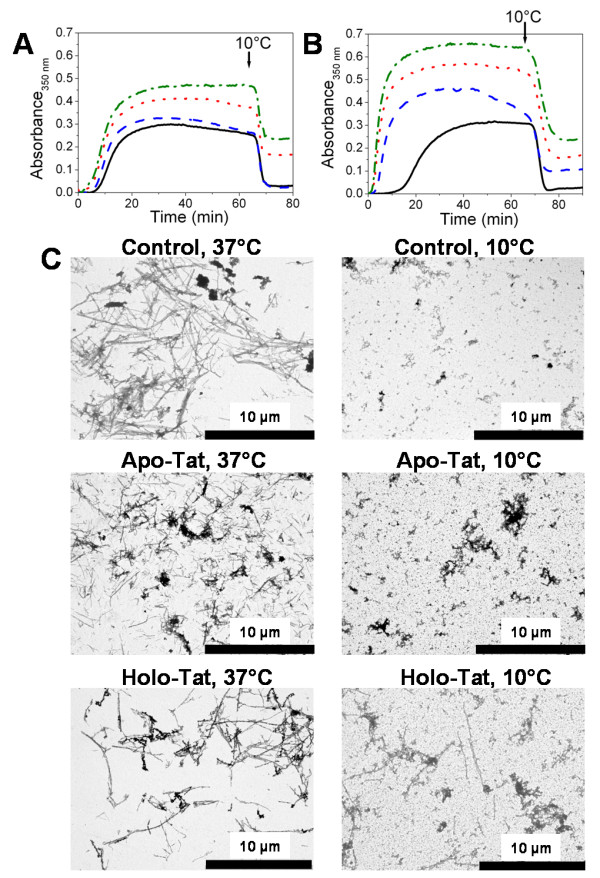
**Effect of Tat on tubulin assembly above the critical concentration (Cr) of tubulin**. A and B. Effect of Tat on tubulin (15 μM) assembly, as measured by turbidimetry at 350 nm. Measurements were performed in the absence (black solid line), or in the presence of 2 μM (blue dashed line), 3 μM (red dotted line), or 4 μM (green dashed-dotted line) of A) apo-Tat or B) holo-Tat, in PMG buffer at 37°C. At the time indicated by the arrow, samples were cooled to 10°C. C. Electron micrographs of 15 μM tubulin in the absence or the presence of 4 μM apo-Tat, or 4 μM holo-Tat at 37°C and after cold depolymerisation at 10°C, in PMG buffer.

Having shown some differences between apo-Tat and holo-Tat with respect to their interaction with tubulin dimers in PG buffer at 20°C, we measured the effects of various concentrations of apo-Tat and holo-Tat on microtubule formation in PMG buffer (20 mM NaPi, 10 mM MgCl_2_, 0.1 mM GTP, 3.4 M glycerol, pH6.5) (Fig. 4). The reactions with 15 μM tubulin were started by warming the samples to 37°C. For the control in the absence of Tat, after a lag time of several minutes, the turbidity increased and reached a plateau (Fig. 4A). Lowering the temperature to 10°C induced a drop in turbidity to its initial values, indicating a total reversibility of the reaction. In the presence of apo-Tat (Fig. 4A) and holo-Tat (Fig. 4B) added at concentrations that have been shown to interact efficiently with microtubules and promote apoptosis in cells [15,16], we observed a shortening of the lag time as well as a strong increase in the rate of assembly and final plateau value. The Tat-induced changes on tubulin assembly were strongly dependent on the protein concentration for both of the Tat forms. At the highest Tat concentration (4 μM), the turbidity plateau was increased by 1.6- and 2.1-fold for apo-Tat and holo-Tat, respectively, as compared with the control plateau value obtained with tubulin alone. Our data obtained with Tat Lai are in line with those previously obtained with Tat HxB2, suggesting that the Tat proteins from both strains exhibit similar activities on tubulin assembly [16].

However, the Tat proteins from the two strains were found to differ in the disassembly step. Indeed, in contrast to Tat HxB2 (Fig. 1A in [16]), when the temperature of the samples was decreased to 10°C, we did not observe a complete disassembly of the microtubules in the presence of both apo-Tat and holo-Tat Lai species. This indicated the presence of cold stable aggregates or polymers with the Tat Lai variant.

To compare further the tubulin assembly induced by the apo- and holo-forms of Tat Lai, the samples were examined by electron microscopy at 37°C at the turbidity plateau and at 10°C, after cold depolymerisation (Fig. 4C). At 37°C, the electron micrographs confirmed the formation of microtubules in the presence of both Tat forms, similar in shape to the controls. However, in addition to microtubules, numerous tubulin aggregates were observed in the presence of apo-Tat. At 10°C, in all conditions (with and without Tat) we observed large rings (outside diameter ≈ 50 nm), likely due to the lack of EGTA in our experiments. Indeed, rings are favored by divalent cations such as Ca^2+ ^[44,45] that are chelated by the EGTA added in the classical buffer used to study microtubule formation [41]. These rings are the main if not, the only observable form in the control. In contrast, we also observed cold stable microtubules in the presence of the holo-Tat (Fig. 4C). With apo-Tat, amorphous tubulin aggregates were observed but microtubules were absent. As a consequence, though the turbidity traces of apo- and holo-Tat forms were similar (Fig. 4A and Fig. 4B), significant differences appear in the nature of the tubulin polymers induced by the two forms of Tat.

**Figure 5:**
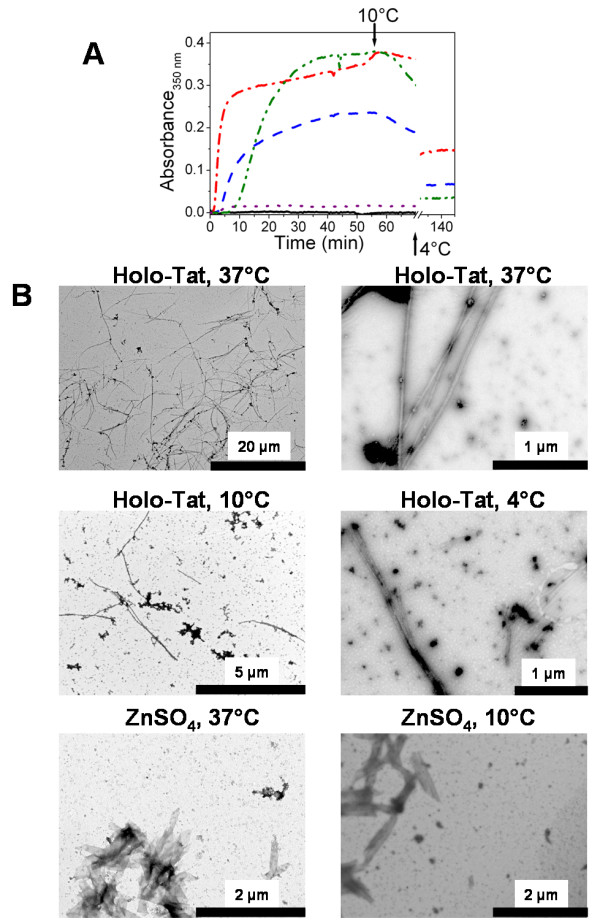
**Holo-Tat promotes and stabilizes microtubules under assembly-conditions, at a tubulin concentration below the critical concentration (Cr)**. A. Effect of Tat on tubulin (6 μM) assembly, as measured by turbidimetry at 350 nm. Measurements were performed in the absence (black solid line), or in the presence of 4 μM holo-Tat (blue dashed line), 8 μM holo-Tat (red dashed-dotted line), 8 μM zinc sulfate (purple dotted line), or 16 μM zinc sulfate (green dashed-dotted-dotted line), in PMG buffer at 37°C. At the time indicated by the first arrow, samples were cooled to 10°C. The second arrow represents one hour of incubation at 4°C. The trace with 8 μM apo-Tat was indistinguishable from the control and was thus not represented. B. Electron micrographs of 6 μM tubulin in the presence of 8 μM holo-Tat, or 16 μM zinc sulfate, in PMG buffer at 37°C and after cold depolymerisation at 10°C or 4°C.

In the next step, the interaction between the different forms of Tat Lai and tubulin were characterized at a tubulin concentration below the critical concentration (Cr), where no tubulin assembly occurs at 37°C (for a review, see [46]). In the absence of Tat, the tubulin Cr value was found to be 9 ± 1 μM, in line with the 8 μM value determined in the presence of EGTA [47]. To be below the Cr, we investigated Tat-tubulin interaction at a 6 μM concentration of tubulin. As for the control (black solid line in Fig. 5A), no significant increase in turbidity was observed when apo-Tat at 8 μM was added at 37°C. In contrast, the same concentration of holo-Tat (8 μM) resulted in a strong increase in turbidity (red dashed-dotted line). This effect was dependent on the holo-Tat concentration, as seen by the different turbidity traces with 4 μM and 8 μM holo-Tat. When the samples were cooled to 10°C, the turbidity slightly decreased but did not fall to zero even after several hours (data not shown). This indicates that a large fraction of the tubulin polymers induced by holo-Tat was stable at 10°C. Further incubation at 4°C during one hour induced a drop of turbidity.

In line with the turbidity data, electron microscopy showed no polymers with tubulin alone or when apo-Tat (See additional file 1) was added to tubulin at 37°C. In contrast, the polymers induced by holo-Tat corresponded to normal microtubules (Fig. 5B). At 10°C, we also observed microtubules. A few stable microtubules were still present after one hour of incubation at 4°C, and were thus responsible for the residual turbidity (Fig. 5A).

The temperature-induced reversibility of tubulin assembly in the presence of holo-Tat indicates that holo-Tat -induced tubulin polymers and tubulin dimers are in equilibrium. This allowed us to calculate a Cr value of 4 ± 1 μM of tubulin in the presence of 8 μM holo-Tat. This Cr value is about two-fold less than the Cr value for tubulin assembly in the absence of holo-Tat.

Since zinc is known to induce tubulin sheets [48-50], we also monitored the effect of zinc on tubulin assembly (Fig. 5A). Only, at the highest zinc concentration (16 μM) that would correspond to a total release of Zn from 8 μM holo-Tat, a strong increase in turbidity was observed (green dashed-dotted-dotted line in Fig. 5A). However, the lag time of this turbidity increase was much longer than the one observed with 8 μM holo-Tat. Moreover, at 8 μM concentration of zinc, which would correspond to a total release of Zn from 4 μM holo-Tat, the effect on turbidity was much weaker than that with 4 μM holo-Tat. In contrast to the microtubules observed in the presence of holo-Tat at all temperatures, tubulin sheets were observed at 37°C and 10°C in the presence of ZnS0_4 _(Fig. 5B). These sheets were no more present at 4°C, in line with the strong drop in turbidity (Fig. 5A).

Thus, the effect of holo-Tat on tubulin assembly can not be attributed to the release of free zinc from holo-Tat. Moreover, these data confirm that in our experimental conditions, two equivalents of zinc are mainly bound to Tat.

Since the 36–48 region of Tat has been previously shown to be necessary and sufficient for the Tat-tubulin interaction [15], we checked whether a Tat(36–48) peptide was able to induce tubulin assembly. Both above and below the Cr, the turbidity traces were indistinguishable from the control ones, even at peptide concentration up to 60 μM (data not shown). This indicates that the 36–48 region is not sufficient to promote microtubule formation.

## Discussion

HIV-1 Tat protein is involved in the weakening of immune defense in AIDS, notably by interacting with microtubules. Several studies showed that Tat from different HIV isolates, and specifically residues 38–72, was able to enhance tubulin assembly *in vitro*, and induce apoptosis via the mitochondrial pathway [14-16]. The efficiency of different Tat variants to promote tubulin assembly was correlated with their efficiency to induce apoptosis and the progression to AIDS [14,16]. However, in these studies, the zinc binding status of Tat was not checked, despite the evidence that Tat is able to bind zinc ions through its cysteine-rich domain *in vitro *[19-21] and that the Tat transactivation function and apoptosis induction seem to depend upon zinc [25,26]. Moreover, mutations of the Cys residues (except Cys^31^) have been shown to impair Tat functions [51], confirming further the relevance of zinc binding in the biological functions of Tat. In addition, the Tat-Oyi variant from highly exposed but persistently seronegative patients has been shown to differ from other Tat variants by a Cys^22^→Ser substitution, which has the consequences of a decrease in the transactivation activity of Tat [52] and Tat-microtubules interaction [16].

In this study, to further understand the importance of zinc in Tat functions, its role on the conformation and the interaction of Tat Lai with tubulin was investigated. Tat Lai was selected since this variant is representative of the subtype B HIV-1 virus, commonly found in infected individuals in Europe and North America [53]. First, we compared the conformations of the apo-form and zinc-bound form of Tat Lai. For the apo-form, an excellent agreement between the diffusion constant measured by FCS and the theoretical diffusion constant of a sphere with the mass of the hydrated Tat protein suggested that the protein was monomeric and poorly folded, in line with the data obtained earlier with other Tat variants [54]. The poor folding of the apo-Tat form was substantiated by the important segmental motion of the Trp^11^-containing domain that prevented the observation of the protein tumbling motion (Table 2). Moreover, the high maximum emission wavelength and complex fluorescence intensity decay of Trp^11 ^suggested that it was well exposed to the solvent and explored a large number of conformations, in agreement with a flexible and poorly folded structure of Tat. This large exposure of Trp^11 ^to the solvent differs, however, from the inclusion of Trp^11 ^in a hydrophobic pocket suggested by the NMR-derived structure of Tat Lai/Bru at pH4.5 [55]. This difference could not be attributed to the oxidation state of Tat since addition of the reducing agent TCEP that prevented oxidation of the -SH groups did not significantly affect any of the measured fluorescence parameters (data not shown). Though a pH-dependent folding involving Trp^11 ^can not be excluded, our data support also recent reports showing that the Trp-containing region is not folded [53,54].

The holo-form of Tat Lai was found to bind two zinc ions through five of its seven cysteine residues, in full agreement with previous results with the Tat(21–38) peptide [21]. The diffusion constant and the mass spectrum of the zinc-bound form strongly suggested that it remains monomeric, in line with most previously published data [6,21,24]. Interestingly, the large solvent-exposure and the complex intensity decay of the Trp^11 ^residue, as well as the absence of a rotational correlation time corresponding to the protein tumbling suggested that the holo-form remains poorly folded. Nevertheless, the increase of the long rotational correlation time (Table 2) suggested a local folding, most likely at the level of the cysteine-rich sequence close to the Trp^11 ^residue. This partial folding is in line with previous observations made with a different variant of Tat [19], suggesting that it may be a general feature in holo-Tat proteins.

Both apo- and holo-Tat were found to promote tubulin assembly at concentrations above the Cr value (9 ± 1 μM in our conditions). Monitoring the assembly by turbidimetry, both Tat forms were found to decrease the initial lag time and increase the rate of assembly. This suggests that both protein forms can promote the nucleation and elongation phases of microtubule formation [46]. Moreover, both forms increased the turbidity plateau by about two-fold over the control (in the absence of Tat). Electron microscopy data as well as the reversibility of the major part of the holo-Tat-induced turbidity increase at 10°C indicate that holo-Tat mainly induces the formation of microtubules. As a consequence, the increase of the turbidity plateau over the control suggests that Tat promotes a larger amount of microtubules than in the control and thus, likely decreases the Cr. This was confirmed by the measured two-fold decrease in the tubulin Cr value induced by holo-Tat (from 9 ± 1 μM to 4 ± 1 μM of tubulin), and the observation of holo-Tat -induced microtubules at a 6 μM tubulin concentration (Fig. 5).

Moreover, the significant fraction of cold-stable microtubules at 10°C further suggests that holo-Tat also prevents microtubule depolymerization. This assumption is strengthened by the observation of cold-stable microtubules after one hour of incubation at 4°C. In the case of the apo-Tat, the turbidity traces were associated with the formation of both microtubules and tubulin aggregates. Since turbidity is a complex function of the number, size and the shape of the scattering particles [56-58], the effect of apo-Tat on the amount of tubulin polymers is difficult to evaluate. Nevertheless, since in contrast to holo-Tat, no microtubules were induced by apo-Tat at a concentration below the Cr, it is likely that apo-Tat marginally affects the Cr value. In addition, the absence of cold-stable microtubules with apo-Tat further suggests that it does not prevent microtubule depolymerization. The cold stabilization of microtubules by only holo-Tat is highly significant, since this cold stabilization *in vitro *has been shown to be representative of the stabilization of the microtubule network in cells [59,60].

The differences between apo-Tat and holo-Tat with respect to tubulin assembly may be partly accounted by their different binding modes to the tubulin dimers. Holo-Tat was found to bind tubulin dimers in discrete complexes while apo-Tat promoted a distribution of tubulin oligomers. In assembly conditions, the discrete complexes with holo-Tat likely nucleate and elongate microtubules more efficiently than control tubulin dimers. Holo-Tat has the same effect than Paclitaxel [61] and Taxotere [62] that also stabilize the microtubules, causing a mitotic block and a subsequent cell death by apoptosis [60], but it remains to be demonstrated that their mechanisms are similar. The tubulin oligomers observed with apo-Tat probably contribute to the formation of tubulin aggregates and microtubules observed in assembly conditions above the Cr. Since oligomers are thought to be precursors for microtubule nuclei [46], their presence may explain the observed increase in the rate of nucleation and elongation in the apo-Tat-promoted assembly of tubulin. Noticeably, the concentration of Tat in our assays was substantially larger than the nM range concentration of Tat in sera of HIV-1-infected patients [63]. However, such Tat concentrations could be locally achieved in lymphoid tissues, where HIV-1 actively replicates [10,63] or within the intracellular medium, as a consequence of efficient internalization of Tat.

Importantly, our data with holo-Tat are fully consistent with the previously reported prevention by cellular Tat of microtubule depolymerization and the concurrent reduction of the level of unpolymerized tubulin in cells [15]. Consequently, holo-Tat likely constitutes the active form of Tat in the cell cytoplasm. By altering the microtubule dynamics, holo-Tat may then lead to a release of the pro-apoptotic Bim protein, leading to apoptosis through the mitochondria pathway [15].

The Tat(36–48) peptide was found to be unable to promote tubulin assembly though this sequence mediates the binding of Tat to tubulin [15]. Since a Tat(38–72) peptide has been previously reported to promote microtubule formation as efficiently as the full-length Tat [16], the 49–72 region of Tat is likely to be required for promoting tubulin polymerization. The basic region of Tat (residues 49–59) is probably important since basic domains play a key role in microtubule-associated proteins [64] by neutralizing a negatively charged region of the tubulin dimer involved in tubulin assembly. The glutamine rich region of Tat (residues 60–72) may be important too, since this region was shown to modulate the binding of Tat to tubulin and the efficiency of Tat in inducing apoptosis [14].

The differences between apo-Tat and holo-Tat in their binding to tubulin dimers and their activation of tubulin assembly are probably a consequence of the limited conformational changes between the two forms. The binding of zinc to the cysteine-rich region and probably to Cys^37 ^most likely modifies the conformation of the ^36^Val-Cys-Phe-Thr^39 ^sequence, which is determinant for binding to tubulin [15]. This conformational change is probably required for the proper positioning of Tat on its tubulin binding site(s) in order to change the assembly properties of tubulin. Large effects on Tat properties resulting from limited conformational changes are not unprecedented since the strong differences in apoptosis induction by Tat proteins from two different strains have also been related to minor structural modifications of Tat [14].

## Conclusion

We demonstrated in this work that the binding of zinc to the Cys-rich region of Tat Lai modulates the protein conformation, most likely by inducing a partial folding. This probably affects the ^36^Val-Cys-Phe-Thr^39 ^region, critical for tubulin binding. This allows Tat to bind tubulin dimers in discrete complexes, while apo-Tat induces oligomers of different sizes. Moreover, holo-Tat but not apo-Tat reduces the Cr and stabilizes the microtubules similarly to intracellular Tat [15], suggesting that holo-Tat is the intracellular active form involved in apoptosis. Inhibition of Tat-induced apoptosis in non infected cells is thought to impair at least in part the loss of immunocompetence provoked by HIV-1 and hopefully convert HIV infection from a progressively immunosuppressive and ultimately fatal disease to a chronic manageable infection. Since the highly conserved cysteine-rich domain of Tat [22,23] likely induces a structure distinct from the eukaryotic zinc fingers, interference with zinc binding to Tat or targeting the binding site of the holo-Tat to tubulin could be promising as new approaches to design antiviral drugs that would not affect the host proteins.

## List of abbreviations

AIDS: Acquired immunodeficiency syndrome; HIV-1: Human immunodeficiency virus type 1; TAR: Trans-acting responsive element; LTR: Long terminal repeat; AUC: Analytical ultracentrifugation; RhB: Rhodamine B; FCS: Fluorescence correlation spectroscopy; TMR: Carboxytetramethylrhodamine.

## Competing interests

The authors declare that they have no competing interests.

## Authors' contributions

CE performed experiments and wrote part of the manuscript. PB participated in the design of the experiments and in the interpretation of the results. PD and EP provided technical support for FCS and fluorescence time-resolved measurements. DA contributed in the design of the experiments. OC and SM synthesized Tat protein and peptides. VP and YM directed the work and finalized the writing of the manuscript. All authors read and approved the final manuscript.

## Supplementary Material

Additional file 1 Electron micrograph of 6 μM tubulin in the presence of 8 μM apo-Tat, in PMG buffer at 37°C. Click here for file
