# Supplementary figures and images for: Modulation of microtubule assembly by the HIV-1 Tat protein is strongly dependent on zinc binding to Tat
Source: Retrovirology. 2008 Jul 9;5:62. doi: 10.1186/1742-4690-5-62 (PMC2483996; doi:10.1186/1742-4690-5-62)

# **Apo-Tat, 37°C**

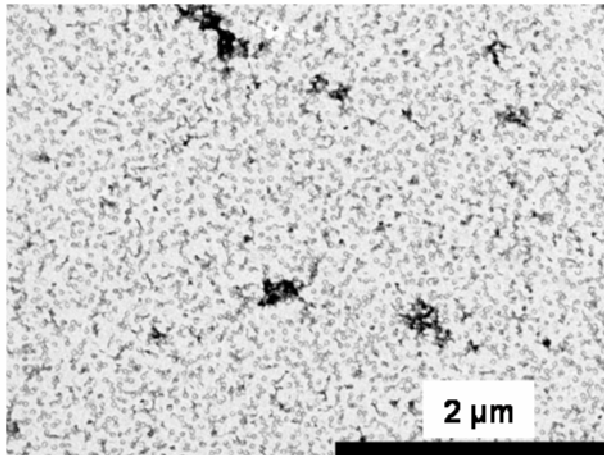

Supplement: Additional file 1 — Electron micrograph of 6 μM tubulin in the presence of 8 μM apo-Tat, in PMG buffer at 37°C. [file 1742-4690-5-62-S1.pdf]
